# Impact of the COVID‐19 Pandemic on the Incidence of Anorexia and Bulimia Nervosa in Dutch Primary Care

**DOI:** 10.1002/eat.24431

**Published:** 2025-03-26

**Authors:** K. Meier, D. van Hoeken, C. C. M. Kager, A. E. van Eeden, A. J. Oldehinkel, H. W. Hoek

**Affiliations:** ^1^ Parnassia Psychiatric Institute The Hague the Netherlands; ^2^ Department of Psychiatry, University Medical Center Groningen University of Groningen Groningen the Netherlands; ^3^ Netherlands Institute for Health Services Research (Nivel), Sentinel Practices Utrecht the Netherlands; ^4^ Department of Epidemiology Mailman School of Public Health, Columbia University New York New York USA

**Keywords:** anorexia nervosa, bulimia nervosa, covid‐19, eating disorders, epidemiology, incidence, primary care

## Abstract

**Objective:**

This study examined the impact of the COVID‐19 pandemic on incidence rates of anorexia nervosa (AN) and bulimia nervosa (BN) in Dutch primary care by comparing pre‐pandemic (2015–2019) and in‐pandemic (2020–2022) periods. We hypothesized that the incidence of AN and BN would increase during the pandemic.

**Method:**

This retrospective cohort study used data from general practitioners' electronic health records from the Nivel Primary Care Database, representing ~0.8% of the Dutch population. Incident AN and BN cases were identified using DSM‐5 criteria. Incidence rates (IR) per 100,000 person‐years were calculated and compared using incidence rate ratios (IRRs).

**Results:**

Overall incidence rates of AN and BN did not increase significantly during the pandemic (AN: IRR = 1.2, 95% CI = 0.8–1.8; BN: IRR = 0.9, 95% CI = 0.5–1.8). Non‐significant increases in AN were observed among females overall (+29%, IRR = 1.3, 95% CI = 0.9–2.0) and among 10‐ to 14‐year‐old females (+28%, IRR = 1.3, 95% CI = 0.4–3.7). The BN incidence decreased significantly among females aged 20–24 years from 53.8 to 10.7 per 100,000 person‐years (IRR = 0.2, 95% CI = 0.0–0.9).

**Discussion:**

Contrary to our hypothesis, we did not find increased incidence rates during the pandemic. The overall incidence of AN among females increased non‐significantly by 29%. The previously observed rising trend in AN among 10‐ to 14‐year‐old females in 1985–2019 did not have a significant sequel into the pandemic era, although it increased by 28%. The BN incidence remained stable overall and decreased significantly among young adult females.

**Summary**

- Concerns have been raised about a potential rise in eating disorders during the COVID‐19 pandemic. Our study, based on data from Dutch general practices, found that the overall incidence of anorexia nervosa increased, but this increase was not statistically significant.
- The overall incidence of bulimia nervosa remained stable.

## 1 Introduction

The COVID‐19 pandemic introduced unprecedented stressors, particularly for individuals at risk for or already experiencing eating disorders (EDs). These stressors included widespread uncertainty and social isolation, reduced access to social support networks, disruptions to daily routines (including limitations on physical activity), and restricted access to mental health services. These factors are known to exacerbate the risk factors for developing an ED (Linardon et al. 2021; Madigan et al. 2025; Meier et al. 2022; Monteleone, Cascino, Barone, et al. 2021; Rodgers et al. 2020; Weissman and Hay 2022). Heightened stress and anxiety during the pandemic may have resulted in a greater perceived loss of control and triggered maladaptive coping mechanisms, such as disordered eating behaviors (Linardon et al. 2021; Rodgers et al. 2020). Increased social media usage and therefore increased exposure to idealized body images on social media may intensify body dissatisfaction, a key ED risk factor (Branley‐Bell and Talbot 2020; Dahlgren et al. 2024; Gilsbach et al. 2022; Rodgers et al. 2020; Schneider et al. 2023). Prolonged isolation may have also reduced access to early intervention, delaying ED diagnosis and treatment. Additionally, fear of contagion and anxiety caused by the pandemic itself likely contributed to the increased risk of ED onset (Linardon et al. 2021; Monteleone, Cascino, Marciello, et al. 2021; Rodgers et al. 2020).

Indeed, emerging evidence indicates that since the onset of and during the COVID‐19 pandemic, there has been a global rise in reported cases of EDs, particularly anorexia nervosa (AN) and bulimia nervosa (BN), along with increased healthcare utilization for EDs (Devoe et al. 2023; Linardon et al. 2021; Madigan et al. 2025; Meier et al. 2022; Rodgers et al. 2020; Solmi et al. 2021). The pandemic has been associated with a 15% increase in ED incidence (Taquet et al. 2022), a 48% increase in hospital admissions (Devoe et al. 2023), and an 11% increase in emergency department visits (Anderson et al. 2022). Furthermore, the severity of ED symptoms among both existing and newly diagnosed ED patients appears to have worsened during the pandemic, as evidenced by longer exercise durations, increased restrictive eating, a greater perceived loss of control, more frequent binge‐eating episodes, and more anxious and depressive symptoms (Gilsbach et al. 2022; Kersten et al. 2023; Meier et al. 2022; Schlegl et al. 2020; Termorshuizen et al. 2020, 2023; Todisco et al. 2023). Previous studies on the impact of COVID‐19 on EDs have been largely conducted in North America, Europe, and Australia, primarily using data from the early pandemic waves (2020–2021). However, a growing body of research, including smaller studies from various countries, suggests similar trends of rising incidence, healthcare visits, and symptom severity worldwide, highlighting the need for urgent changes in ED policy, practice, and research (Cooper et al. 2020; Holmes et al. 2020; Meier et al. 2022; Solmi et al. 2021; Todisco et al. 2023).

The patterns of identification and management of EDs in primary care settings offer a unique perspective on the pandemic's effects. This data source has been largely overlooked, with many studies focusing instead on emergency department visits and hospital data (Madigan et al. 2025). Although emergency department and hospital data primarily capture severe cases—potentially underrepresenting milder presentations, hospitals in certain healthcare systems (such as the U.S.) also serve as critical points of care for uninsured or underinsured individuals with limited access to primary care. Given that primary care serves as the first and ongoing point of contact for most patients, it captures a broader spectrum of cases, including early‐stage presentations, thereby reducing selection bias. However, primary care data also introduces potential biases, as GPs may be less likely to screen for or diagnose EDs compared to specialists, potentially leading to underreporting of cases.

Few studies comparing ED cases in primary care before and during the pandemic have yielded inconclusive results (Madigan et al. 2025). An analysis of over 10 million electronic records of UK general practitioner (GP) contacts found a reduction in contact behavior for EDs and other mental health conditions when comparing pre‐ and in‐pandemic periods, following population‐wide restriction measures (Mansfield et al. 2021). Similarly, a large Belgian register‐based study noted a decrease in GP visits for EDs during the pandemic (Vandamme et al. 2023). Three other large studies observed an increase in GP visits for ED among children and adolescents during the pandemic, compared to before (Bittner Gould et al. 2022; Raventos et al. 2023; Surén et al. 2022). Mixed results observed in recent studies (Madigan et al. 2025) may stem from differences in ED management across primary care practices, underreporting, variations in case definitions, the shift to telehealth services, and changes in primary care accessibility during the pandemic.

The COVID‐19 pandemic in the Netherlands involved multiple lockdowns and fluctuating public health measures aimed at controlling infection rates. Between March 2020 and early 2022, strict lockdowns, social distancing mandates, and the closure of schools, gyms, and non‐essential businesses were implemented. A nationwide curfew was enforced from January to April 2021, further limiting social interactions and mobility (Government of the Netherlands n.d.). These measures, alongside high infection peaks in late 2020 and early 2022 (Government of the Netherlands n.d.), significantly disrupted daily life.

In the Netherlands, GPs act as ‘gatekeepers’ to specialized medical services, meaning that patients cannot directly access secondary or tertiary care without first consulting their GP. Therefore, primary care is the initial point of formal detection of EDs in the Dutch health care system. This minimizes referral bias in incidence studies in Dutch GP settings—an essential factor when comparing incidence studies of EDs. Moreover, Dutch GPs are notified when a patient is admitted to or discharged from a hospital, as well as when outpatient treatments or consultations take place. This ensures that Dutch GPs are always informed when an ED diagnosis is later confirmed or established. Every resident of the Netherlands has guaranteed access to primary care because of the introduction of a universal mandatory basic health insurance in 2006.

Given the central role of GPs, primary care in the Netherlands is an ideal setting for studying the incidence of EDs (Smink et al. 2012). Previously, our research team found that the overall incidence of AN has remained relatively stable over the pre‐pandemic period from 1985 up to 2020 in Dutch general practice (Hoek 1991; Hoek et al. 1995; Van Son et al. 2006; Smink et al. 2016; Van Eeden et al. 2023). Interestingly, over this same period, the incidence of AN among 10‐14‐year‐old females showed a significant rise from 8.6 to 38.6 per 100,000 person‐years (Van Eeden et al. 2023). The incidence of BN declined from the late 1980s to the 2000s and then stabilized from 2010 onward (Hoek 1991; Hoek et al. 1995; Van Son et al. 2006; Smink et al. 2016; Van Eeden et al. 2023, 2021).

The current study examines the impact of the COVID‐19 pandemic on incidence rates of AN and BN in Dutch primary care settings by comparing the 2015–2019 (pre‐pandemic) and 2020–2022 (in‐pandemic) periods. We hypothesized that the incidence of both AN and BN would increase during the pandemic compared to the pre‐pandemic period. Because of notable findings for the group of 10‐ to 14‐year‐old females in our previous pre‐pandemic study, we were specifically interested in the data on 10‐ to 14‐year‐old females with AN.

## 2 Method

### 2.1 Sample

We used data from the Nivel (the Netherlands Institute for Health Services Research) Primary Care Database (Nivel‐PCD) (Nivel n.d.). This is a register containing health care information routinely recorded by (among other primary care providers) GPs who participate in the Nivel Sentinel Practices network in the Netherlands. The Nivel‐PCD has been in place since 1970, with participating GPs continuously registering data on health problems and treatments of their patients. They have registered data on EDs since 1985.

Each year, approximately 40 general practices, comprising around 70 GPs, contribute to the database. The GPs served approximately 1% of the Dutch population during the 1980s and 1990s, and 0.8% of the population in subsequent years. Although our data reflect a subset of the Dutch population, participating GPs are selected by the Nivel based on the regional location and population density of their practices, ensuring coverage that reflects the Dutch population's distribution by sex, age, region, and population density (Donker 2016, 2018a, 2018b, 2019; Jansen et al. 2022).

### 2.2 Procedure

GPs from the Nivel Sentinel Practices network considered whether each patient who consulted them might be suffering from an ED. The recording of an International Classification for Primary Care (ICPC) code (World Health Organization 2003) for a suspected ED in the GP's electronic patient record system prompted the GP to fill out an online form, documenting the patient's sex, age, ED‐related symptoms, height, weight, coexisting medical conditions, referral details to specialized care, and the date when the ED was first identified by any healthcare professional, including the GP. Data on gender, race, and ethnicity were not collected. Nivel provided these electronic forms to the research team in a pseudonymized data file containing records from the preceding year. The questionnaire procedure ensures that a maximum of one questionnaire per patient is generated per year (365 days). A pseudonymized linkage table is used to connect earlier questionnaires belonging to the same patient. Cases were considered incident if the first registration of an ED occurred during the study period. If a previously reported incident case was reported again within 5 years of the initial mention, it was classified as a prevalent case. The same case identification procedures were followed as in our team's previous publications on the Nivel data on EDs (Hoek 1991; Hoek et al. 1995; Van Son et al. 2006; Smink et al. 2016; Van Eeden et al. 2023), with the exception that we now employed DSM‐5 instead of DSM‐IV diagnostic criteria. Since 2015, feeding and eating disorder diagnoses that were first introduced in the DSM‐5 (binge eating disorder [BED], pica, rumination disorder [RD], avoidant/restrictive food intake disorder [ARFID], other specified feeding or eating disorder [OSFED], and unspecified feeding or eating disorder [UFED]) have been registered in the practices as well. However, this study focuses on AN and BN only to ensure comparability with previous Nivel‐PCD research.

The total study period was divided into a pre‐pandemic (2015–2019) and an in‐pandemic (2020–2022) period. The inclusion criterion was a new ED diagnosis according to DSM‐5 in the period 2015–2022. Although all DSM‐5 EDs were registered, we focused on AN and BN. Our research team of ED experts evaluated whether registrations of potential EDs provided by the GPs met DSM‐5 diagnostic criteria for an ED. Since the start of the Nivel cohort, the same psychiatrist (senior author HWH) has consistently been responsible for classifying the ED cases, ensuring continuity and consistency in diagnostic decisions.

Previous studies (Hoek 1991; Hoek et al. 1995; Van Son et al. 2006; Smink et al. 2016; Van Eeden et al. 2023) included a two‐year extension of the search period to identify cases that were incident during the study period but whose registration was delayed due to administrative reasons. These delays occur when diagnoses, such as an ED, are documented after the study period due to delayed communication—for instance, when an ED diagnosis is mentioned in a letter from specialized healthcare services, which are typically sent to the patient's GP on an annual basis. Because 2024 data were not available at the time of writing, in our study we used a one‐year extension period to ensure methodological consistency across the two periods in our study. Thus, 2020 data were reviewed for delayed registered incident cases from the pre‐pandemic period (2015–2019), and 2023 data were reviewed for delayed incident cases for the in‐pandemic period (2020–2022).

### 2.3 Statistical Analyses

Incidence rates (IRs) were calculated overall, for females, and for females per 5‐year age category, for both the pre‐pandemic (2015–2019) and the in‐pandemic (2020–2022) periods. These subgroups were chosen to maintain consistency with the methodology of prior research utilizing this database, thereby ensuring the comparability of findings. IRs were calculated by dividing the number of new cases by the total number of person‐years in the Dutch sentinel practices, expressed per 100,000 person‐years. We calculated the percentage of patients who were referred to specialized mental health care. The inclusion of 1 in the confidence interval (CI) indicates that the result is not statistically significant at the *p* < 0.05 level. To evaluate changes in IR between the two periods, incidence rate ratios (IRRs) were computed by comparing the incidence rates of the in‐pandemic period (2020–2022) with those of the pre‐pandemic period (2015–2019). Statistical significance was assessed using a 95% CI, and significance levels were set at *p* < 0.05. All analyses were performed using R.

### 2.4 Ethical Considerations

Participating GPs are contractually required to inform their patients about their participation in the Nivel‐PCD and about the patients’ right to opt out of data inclusion in the database. Data are pseudonymized before leaving the practices and do not contain any directly identifying personal information. The study meets the requirements for exemption from approval by a medical ethics committee under the Dutch Medical Research Involving Human Subjects Act (WMO) and complies with the Dutch Medical Treatment Act (WGBO) and the European Union General Data Protection Regulation (GDPR). The use of electronic health records for research purposes is allowed under certain conditions. When these conditions are fulfilled, neither obtaining informed consent from patients nor approval by a medical ethics committee is required for this type of observational study containing no directly identifiable data (art. 24 GDPR Implementation Act jo Art. 9.2 subj GDPR).

## 3 Results

For the pre‐pandemic period (2015–2019), using a one‐year extension to identify late registrations, a total of 214 potential ED cases were reported. Of these, 87 were excluded because they were prevalent (pre‐existent) ED cases (*n* = 83) or did not have an ED (*n* = 4). Furthermore, 41 incident cases with another ED (BED, pica, RD, ARFID, OSFED, and UFED) were also excluded from the study. The resulting five‐year pre‐pandemic study cohort contained 86 incident AN or BN cases according to DSM‐5: 58 with AN (including 4 males) and 28 with BN (including 2 males).

During the in‐pandemic period (2020–2022), GPs reported a total of 153 potential ED cases. Of these, 80 were excluded because they were prevalent (pre‐existent) ED cases (*n* = 65) or did not have an ED (*n* = 15). Furthermore, 22 incident cases with another ED (BED, ARFID, or OSFED) were also excluded. The resulting in‐pandemic study cohort contained 51 incident AN or BN cases according to DSM‐5: 37 with AN (all female) and 14 with BN (all female).

### 3.1 Anorexia Nervosa

The overall IR of AN did not differ significantly between the pre‐pandemic and in‐pandemic periods (IRR = 1.2, 95% CI = 0.8–1.8), with a pre‐pandemic IR of 8.1 (95% CI = 6.2–10.5) and an in‐pandemic IR of 9.8 (95% CI = 6.9–13.5) per 100,000 person‐years (see Table 1). The mean age at detection was similar when comparing the pre‐pandemic (mean = 20.2, SD = 8.1) and in‐pandemic (mean = 20.0, SD = 5.9) cohorts. Among females, the overall incidence of AN increased by 29%, from 15.1 per 100,000 person‐years during the 2015–2019 period to 19.5 per 100,000 person‐years in 2020–2022 (IRR = 1.3, 95% CI = 0.9–2.0). Looking specifically at 10‐ to 14‐year‐old females, an increase of 28% was observed, from 38.6 per 100,000 person‐years during the 2015–2019 period to 49.4 per 100,000 person‐years in 2020–2022 (IRR = 1.3, 95% CI = 0.4–3.7). None of the differences in IR between specific sex or age groups were significant when comparing the two study periods. The IRs of AN are presented in Table 1. During the in‐pandemic period, 81% of AN patients were referred to specialized mental healthcare, whereas 74% were referred in the pre‐pandemic period.

**TABLE 1:** Incidence of anorexia nervosa (DSM‐5) per 100,000 person‐years.

Study period	2015–2019				2020–2022				IRR	IRR 95% CI
Females	*N*	PY	IR	95% CI	*N*	PY	IR	95% CI		
Age range										
5–9	0	19,309	—	—	1	11,724	8.5	0.2–47.6	—	—
10–14	8	20,745	38.6	16.7–76.0	6	12,146	49.4	18.1–107.5	1.3	0.4–3.7
15–19	22	21,254	103.5	64.9–156.7	12	13,415	89.5	46.2–156.2	0.9	0.4–1.7
20–24	14	22,316	62.7	34.3–105.2	9	18,799	47.9	21.9–91.0	0.8	0.3–1.8
25–29	4	24,307	16.5	4.5–42.1	8	21,756	36.8	15.9–72.5	2.2	0.7–7.4
30–34	2	22,604	8.9	1.1–32.0	0	18,552	—	—	—	—
35–64	4	141,140	2.8	0.8–7.3	1	92,932	1.1	0.0–6.0	0.4	0.0–3.4
Overall females	54	358,143	15.1	11.3–19.7	37	189,323	19.5	13.8–26.9	1.3	0.9–2.0
Overall males + females	58	713,910	8.1	6.2–10.5	37	376,398	9.8	6.9–13.5	1.2	0.8–1.8

Abbreviations: 95% CI, 95% confidence interval; IR, incidence rate; IRR, incidence rate ratio; *N*, number of cases; PY, person‐years.

### 3.2 Bulimia Nervosa

The overall IR of BN did not differ significantly between the pre‐pandemic and in‐pandemic periods (IRR = 0.9, 95% CI = 0.5–1.8), with a pre‐pandemic IR of 3.9 (95% CI = 2.6–5.7) and an in‐pandemic IR of 3.7 (95% CI = 2.0–6.2) per 100,000 person‐years (see Table 2). The mean age at detection was similar when comparing the pre‐pandemic (mean = 26.5, SD = 9.2) and in‐pandemic (mean = 26.9, SD = 10.0) cohorts. There was a significant decrease in IR by 80% (IRR = 0.2, 95% CI = 0.0–0.9) for the 20‐ to 24‐year‐old females when comparing the pre‐pandemic (IR = 53.8, 95% CI = 27.8–93.9) and in‐pandemic (IR = 10.7, 95% CI = 1.3–38.5) periods. None of the differences in IR between other specific sex or age groups were significant when comparing the two study periods. The highest pre‐pandemic IR was found in young adult females aged 20–24 years, whereas the highest in‐pandemic IR was found in adolescent females aged 15 to 19 years. For a detailed overview of the IRs of BN, see Table 2. During the pandemic period, 86% of BN patients were referred to specialized mental healthcare, whereas 79% were referred in the pre‐pandemic period.

**TABLE 2:** Incidence of bulimia nervosa (DSM‐5) per 100,000 person‐years.

Study period	2015–2019				2020–2022				IRR	IRR 95% CI
Females	*N*	PY	IR	95% CI	*N*	PY	IR	95% CI		
Age range										
5–9	0	19,309	—	—	0	11,724	—	—	—	—
10–14	0	20,745	—	—	1	12,146	8.2	0.2–45.9	—	—
15–19	4	21,254	18.8	5.1–48.2	4	13,415	29.8	8.1–76.3	1.6	0.4–6.3
20–24	12	22,316	53.8	27.8–93.9	2	18,799	10.7	1.3–38.5	0.2	0.0–0.9
25–29	3	24,307	12.3	2.5–36.1	2	21,756	9.2	1.1–33.2	0.7	0.1–4.5
30–34	3	22,604	13.3	2.7–38.8	1	18,552	5.4	0.1–30.1	0.4	0.0–3.9
35–64	4	141,140	2.8	0.8–7.3	4	92,932	4.3	1.2–11.0	1.5	0.4–6.1
Overall females	26	358,143	7.3	4.7–10.6	14	189,323	7.4	4.0–12.4	1.0	0.5–2.0
Overall males + females	28	713,910	3.9	2.6–5.7	14	376,398	3.7	2.0–6.2	0.9	0.5–1.8

Abbreviations: 95% CI, 95% confidence interval; IR, incidence rate; IRR, incidence rate ratio; *N*, number of cases; PY, person‐years.

## 4 Discussion

This study aimed to investigate the IRs of AN and BN in primary care in the Netherlands by comparing the in‐pandemic period (2020–2022) with the pre‐pandemic period (2015–2019).

Contrary to our hypothesis, there was no significant increase in the overall incidence of both AN and BN across the two periods. Although the incidence of AN among girls aged 10–14—a group of particular interest due to prior findings of a substantial pre‐pandemic increase over the past four decades—further increased, this increase was also not significant. No significant differences were found in the incidence of AN and BN across other sex and age groups either, except for BN among individuals aged 20–24, which showed a significant decrease rather than an increase from the pre‐pandemic to the in‐pandemic period.

Several factors may account for the absence of the expected increases in overall incidence of AN and BN. First, the COVID‐19 pandemic significantly altered primary care services in the Netherlands. Although, in response, e‐health and teleconsultation options quickly became available (Lambooij et al. 2022; Van der Vaart et al. 2022), many patients still avoided seeking help due to fear of infection, concerns about burdening the healthcare system, and perceptions that their symptoms were not severe enough to warrant a visit (Homburg et al. 2022; Lambooij et al. 2022; Termorshuizen et al. 2023). However, face‐to‐face medical evaluations are crucial for the early detection of ED patients and for the identification of those with medical instability who may need hospital admission (Couturier et al. 2021; Meier et al. 2022). Between March and June 2020 (during the first wave of the pandemic), GPs in the Netherlands experienced an 11% decline in patient visits, even among those with urgent health concerns (Lambooij et al. 2022). There was a significant increase in short consultations, often via telephone or email, which continued beyond the first wave (Lambooij et al. 2022). Between September and December 2020 (during the second wave of the pandemic), GP consultations returned to 2019 levels, suggesting that missed consultations from the first wave were not compensated for (Lambooij et al. 2022). Consequently, the reduced in‐person consultations may have delayed the detection and treatment of new ED cases, affecting timely intervention and appropriate care. Additionally, individuals with mild ED symptoms who would otherwise have sought help but did not do so during the pandemic may have recovered without medical intervention and gone unnoticed. In 2021, compared to 2020, fewer Dutch individuals with EDs reported being actively ill, and more reported experiencing lingering ED symptoms (Termorshuizen et al. 2023). This pattern was not observed in Sweden and the United States, where the distribution of individuals reporting being actively ill, experiencing lingering symptoms, or no symptoms did not change over time (Termorshuizen et al. 2023). Overall, the disruptions and adaptations in primary care likely played a significant role in shaping ED diagnosis patterns during this period. This may lead to a backlog of undiagnosed ED cases that could emerge over time (Homburg et al. 2022; Termorshuizen et al. 2023).

A second possible explanation for our findings relates to the increased complexity and severity of EDs during the pandemic. Many studies have noted that during the pandemic, pre‐existing ED patients who sought care often had more severe symptoms and more complex problems (Gilsbach et al. 2022; Kersten et al. 2023; Schlegl et al. 2020; Termorshuizen et al. 2020, 2023; Todisco et al. 2023). Due to the avoidance of seeking help, the health conditions of some new ED cases may have deteriorated to the point of emergency. In our study, we observed an overall referral rate to secondary care of 82% during the pandemic, compared to 63% in 1985–1989 and 76% in 2015–2019 (Van Eeden et al. 2023). Increases in IRs for AN and BN observed in secondary care during the pandemic may reflect increased referral rates rather than a true rise in incidence, potentially suggesting that cases identified during the pandemic were more severe. However, seeking care directly from specialized healthcare providers, without involving primary care, would not necessarily reduce the number of new diagnoses recorded in primary care data, as Dutch GPs are generally required to be informed by other healthcare professionals about their patients' diagnoses. In such cases, bypassing primary care might lead to delayed registration of cases in the Nivel‐PCD, rather than a complete omission.

Although our study highlights healthcare disruptions and lifestyle changes as potential factors influencing ED incidence, it is equally plausible that COVID‐19 had no measurable impact. Supporting this hypothesis, our data indicate no significant changes in AN or BN overall. Although several studies suggest that the pandemic heightened the risk of developing an ED in some respects (Linardon et al. 2021; Monteleone, Cascino, Barone, et al. 2021; Rodgers et al. 2020), it may have also mitigated certain triggers, which could have lowered the risk. For young adults transitioning to higher education or the workforce, university closures and remote work led many to remain at home, benefiting from increased family support, parental supervision, and regular meal patterns (Termorshuizen et al. 2020). Healthcare access may also have played a role. Individuals with milder ED symptoms who might have sought care pre‐pandemic may have delayed or avoided it due to healthcare disruptions. Consequently, while our data suggest stable AN and BN incidence, these figures likely underestimate the true incidence in the population, as undetected mild cases may not have been registered in primary care. Meanwhile, the average severity of detected cases may have increased, as those who did seek care during the pandemic were more likely to have more severe symptoms, as reported in several studies (Gilsbach et al. 2022; Kersten et al. 2023; Schlegl et al. 2020; Termorshuizen et al. 2020, 2023; Todisco et al. 2023).

Although the overall incidence of AN and BN remained stable during the pandemic, we observed an upward trend in AN incidence among young adolescent girls (10–14 years) and a significant decrease in BN incidence among young adult women (20–24 years). One could speculate that these trends reflect different underlying mechanisms. For some, a perceived loss of control triggered by the lockdowns may have led to restrictive eating behaviors as a way to regain control, contributing to AN. For others, changes in daily routines—as well as moving back in with family or reduced access to triggering environments like food stores and restaurants—may have disrupted binge‐eating patterns, potentially contributing to the decline in BN. Additionally, fewer in‐person social interactions may have reduced direct social comparison; however, increased exposure to social media during the pandemic could have counteracted this effect. These interpretations remain speculative, and alternative explanations—including potential biases in healthcare‐seeking behavior and diagnosis—should be considered.

### 4.1 Anorexia Nervosa

Compared to the 2015–2019 data in our previous publication (Van Eeden et al. 2023), there were two fewer cases of AN due to the employment of a one‐year (2020) extension period for detection of incident cases as opposed to the two‐year (2020–2021) extension period previously employed. The number of AN cases for 2015–2019 in the current study was unaffected by our change to DSM‐5 diagnostic criteria, however. This previous research from our study group (Van Eeden et al. 2023) showed that over the decades preceding the pandemic (1985–2019), the global incidence of AN had remained relatively stable, but that there was a significant rise in the incidence of AN among girls aged 10–14 years in primary care in the Netherlands, increasing from 8.6 to 38.6 per 100,000 person‐years. In the current study, we observed a further 28% increase in this group, to 49.4 per 100,000 person‐years in 2020–2022. Although this finding did not reach statistical significance, it extends the previously identified increasing trend. Furthermore, unlike the previous study (Van Eeden et al. 2023), our current findings indicate an upward trend across all age groups. This suggests that pandemic‐related stressors have a more overarching, widespread impact on eating behaviors and mental health than what AN triggers in non‐pandemic periods. An extended observation period is required to determine whether this is a temporary increase due to the COVID‐19 pandemic or a consistent development.

The ongoing rise in the incidence of AN among 10‐ to 14‐year‐old girls during the pandemic is a worrisome trend. Early adolescence is a critical developmental period, marked by heightened sensitivity to body image, and a peak period for the onset of EDs (Breton et al. 2022). The rise in AN incidence among adolescents in recent years may be closely linked to the growing use of social media. Social media usage has been steadily increasing over the past decade, becoming an integral part of daily life among youth (Anderson and Jiang 2018; Council on Communications and Media 2016; Netwerk Mediawijsheid 2021) and leading to more mental health problems among young (pre‐)adolescents (e.g., see Council on Communications and Media 2016; McCrae et al. 2017; Riehm et al. 2019). During the COVID‐19 pandemic, social media use surged even more due to increased social isolation, school closures, and the need for alternative means of communication and entertainment (Netwerk Mediawijsheid 2021; Statista 2021, 2023). Social media platforms promote idealized and unrealistic body standards, an issue compounded by algorithms that repeatedly suggest appearance‐focused content based on users' prior interactions. A core feature of AN is the distorted perception of body weight or shape, which disproportionately impacts an individual's self‐worth and self‐assessment. The constant exposure to, and comparison with, unattainable appearance ideals can negatively impact body image, foster body dissatisfaction, and increase the risk of disordered eating behaviors and EDs (Dahlgren et al. 2024; Padín et al. 2021; Saul et al. 2022). This effect is particularly pronounced on visual social media platforms, such as TikTok and Instagram (Saiphoo and Vahedi 2019). Adolescent girls may be more vulnerable to the adverse effects of social media on eating cognitions and behaviors than boys, as societal appearance ideals and beauty standards disproportionately target women and girls, reinforcing pressures to attain unrealistic body shapes and sizes (Dahlgren et al. 2024). However, it is important to note that the observed rising trend in AN incidence was not statistically significant and was not exclusive to 10‐ to 14‐year‐old girls, as similar increases were observed in other age groups, such as 25‐ to 29‐year‐olds. Therefore, the proposed explanations should be interpreted with caution, as they remain hypothetical.

### 4.2 Bulimia Nervosa

The employment of a one‐year (2020) extension period for detection of incident cases in the 2015–2019 cohort as opposed to the two‐year (2020–2021) extension period previously employed (Van Eeden et al. 2023) resulted in one fewer case of BN in the current study. However, our change to DSM‐5 diagnostic criteria raised the number of BN cases for 2015–2019 in the current study. Our previous research had shown that over the decades preceding the pandemic (1985–2019) the overall incidence of BN decreased significantly before leveling off in the 2010s (Van Eeden et al. 2023). Within the current study, the overall incidence of BN did not change significantly, which aligns with the stabilization observed in the 2010s and indicates continuity in the previous trend. We did observe a significant decrease in the incidence of BN among young adult females aged 20 to 24 years from 53.9 to 10.9 per 100,000 person years when comparing the in‐ and pre‐pandemic periods, however. The increased accessibility of food while staying at home may have had adverse effects on individuals with binge eating (Weissman and Hay 2022). However, the increased time at home may have alleviated some of the negative consequences caused by the pandemic. Greater parental support and connection with family, reduced pressure to engage in social activities, and more time for self‐care potentially mitigated some of the behaviors associated with EDs in this age group (Termorshuizen et al. 2020). The increased parent–child closeness during COVID‐19 home confinement may have facilitated earlier detection of binge eating behaviors by family members, both due to heightened personal attention and the reduced freedom to engage in binge episodes. As for AN, an extension of the observation period is required to determine whether this is a temporary fluctuation or a consistent development.

### 4.3 Strengths and Limitations

Our study has several strengths, including a large, representative cohort covering 0.8% of the Dutch population. The use of primary care‐based data allowed us to capture a broad range of patients of all ages, including those who did not progress to secondary or tertiary care, thereby minimizing referral bias—an essential factor when comparing incidence studies of EDs. Our study employed a methodology consistent with previous studies from our research team on the incidence of AN and BN in primary care. The reliability of our analyses was strengthened by consistently applying uniform case definitions and the involvement of the same psychiatrist for final diagnostic decisions across four decades. Using DSM‐5 criteria ensured alignment with current diagnostic standards. Additionally, the use of an extended questionnaire completed by GPs provided more detailed and comprehensive data for diagnostic assessments, strengthening the robustness of our findings compared to other incidence studies (Bittner Gould et al. 2022; Mansfield et al. 2021; Surén et al. 2022; Vandamme et al. 2023).

There are also several limitations to consider. First, our IRs are likely underestimates of the true incidence in the population. IRs in primary care settings are generally lower than in population‐level studies due to two selection filters: the patient's decision to consult a GP—often negatively influenced by denial, stigma, unawareness of the disorder, and in this study pandemic‐related concerns—and the GP's competence to detect the disorder (Huxley et al. 2023; Van Eeden et al. 2021). Despite efforts to enhance detection by participating GPs through oral and written training, some ED cases may have been missed. As cases of AN are generally more easily detected due to the visible underweight status compared to BN, IRs for AN might be closer to the population IR than those for BN. Furthermore, it is possible, though unlikely, that some GPs occasionally forgot to fill out the information sheet and document a case, which could have further contributed to underestimation.

Second, no male ED incident cases were recorded during the in‐pandemic period, and only a few were recorded during the pre‐pandemic period. Some male cases may have been missed by GPs due to the dual stigma of EDs as both mental and “female‐specific” disorders. There generally is a paucity of research on men with EDs, leading to an incomplete understanding of their incidence, diagnosis, and treatment needs.

Third, our IRs are based on the time of detection rather than the onset of the ED, a common issue in ED research due to delays in patients seeking help (Huxley et al. 2023). Although the discrepancy between the age at onset and the age at diagnosis may influence the age distribution observed in our data, it is unlikely to alter the overall IRs.

Fourth, despite our large cohort, the limited number of cases reduced the statistical power to detect small but clinically significant trends. The low number of cases identified reflects the rarity of diagnosed EDs in the general population. However, the observed IRs align with expected population rates, suggesting that our findings are representative of the ED occurrence in Dutch primary care.

Fifth, a major limitation of this study is the exclusion of other EDs, such as BED. Although other EDs have been registered in Nivel‐PCD since the introduction of DSM‐5 in 2015, their incidences have remained low due to the study's original focus on AN and BN and the historical emphasis on these disorders in GP training. Additionally, our pre‐pandemic study period was structured to align with prior research that exclusively analyzed AN and BN. Future studies using larger datasets or alternative healthcare settings may provide a more comprehensive understanding of incidence trends across all ED subtypes.

Another limitation of this study is the binary categorization of sex in the dataset, which does not account for gender diversity.

Finally, our findings reflect the situation in the Dutch population and may not be generalizable to countries with different sociodemographic profiles, healthcare systems, or COVID‐19 (protective) measures.

## 5 Conclusion

This study found no significant change in the incidence rates of AN and BN in Dutch general practice from the pre‐ to the in‐COVID‐19 pandemic period, where an increase in incidence was expected. There was a decrease in BN incidence among young adults aged 20–24 years. Notably, the increase in incidence of AN among females aged 10–14 years that was observed over the period 1985–2019 did not have a significant sequel into the pandemic era, although we did observe a further, though non‐significant, 28% increase in incidence.

## Author Contributions

**K. Meier:** conceptualization, data curation, formal analysis, methodology, writing – original draft, writing – review and editing. **D. van Hoeken:** methodology, supervision, writing – review and editing. **C. C. M. Kager:** data curation, writing – review and editing. **A. E. van Eeden:** conceptualization, writing – review and editing. **A. J. Oldehinkel:** supervision, writing – review and editing. **H. W. Hoek:** conceptualization, methodology, supervision, writing – review and editing.

## Ethics Statement

The study meets the criteria for exemption from approval by a medical ethics committee under the Dutch Medical Research Involving Human Subjects Act (WMO) and complies with the Dutch Medical Treatment Act (WGBO) and the European Union General Data Protection Regulation (GDPR).

## Conflicts of Interest

The authors declare no conflicts of interest.

## Data Availability

The data that support the findings of this study are available on reasonable request from the corresponding author. The data are not publicly available due to privacy regulations and ethical restrictions related to patient confidentiality.
